# Sacrohysteropexy performed as uterus conserving surgery for pelvic organ prolapse: Review of case files

**DOI:** 10.12669/pjms.325.10307

**Published:** 2016

**Authors:** Ayesha Khan, Riffat Jaleel, Farah Deeba Nasrullah

**Affiliations:** 1 Prof. Ayesha Khan, FRCOG. Professor & Head of Department, Department of Obstetrics & Gynaecology, Unit I, Dow University of Health Sciences, Karachi, Pakistan; 2 Dr. Riffat Jaleel, FCPS. Associate Professor, Dow Medical College & Civil Hospital Karachi, Department of Obstetrics & Gynaecology, Unit I, Dow University of Health Sciences, Karachi, Pakistan; 3 Dr. Farah Deeba Nasrullah, MCPS, FCPS. Assistant Professor, Dow Medical College & Civil Hospital Karachi, Department of Obstetrics & Gynaecology, Unit I, Dow University of Health Sciences, Karachi, Pakistan

**Keywords:** Sacrohysteropexy, Utero vaginal prolapse, Pelvic organ prolapse

## Abstract

**Objective:**

To assess the outcome and safety of sacrohysteropexy as uterus conserving surgery for pelvic organ prolapse in young women and to assess patients’ satisfaction with the procedure.

**Methods:**

This is a case series of patients operated at Sind Government Lyari General Hospital and Civil Hospital Karachi, between January, 2007 to October, 2015. Data of the patients who had sacrohysteropexy were reviewed. Complications during surgery and post-operative period including haemorrhage, visceral injury, paralytic ileus and peritonitis were studied. Success of procedure, need of blood transfusion, hospital stay and condition on discharge and six weeks follow-up were noted. Data were analyzed using SPSS version 16. Mean ± SD was calculated for numerical, while frequencies were computed for categorical variables.

**Results:**

Data of 60 patients were reviewed. Early post-operative success was 100%. Duration of surgery was less than two hours in 57 (95%) patients. Blood loss was negligible in majority of cases. Out of all 60 cases, 52 (86.7%) did not suffer any complication. One patient had ureteric injury, while one patient sustained bowel injury. Two patients had paralytic ileus. Four patients suffered from abdominal wound infection. All patients were managed satisfactorily. Mean duration of stay in hospital was four days. Upon follow up 96.7% patients were satisfied with results of operative procedure. Sixteen (26.7%) patients complained of backache on follow-up visit.

**Conclusion:**

This review concludes that sacrohysteropexy was successful in all cases in early post-operative period. It is a safe procedure and should be considered as an option for the treatment of pelvic organ prolapse in young women, in whom uterine conservation is required.

## INTRODUCTION

Pelvic organ prolapse (POP) is the descent of the pelvic organs including bladder, rectum, uterus, vaginal vault and intestines, beyond their anatomical confines. POP is very common in pre and postmenopausal women and affects around half of all parous women. In a study from USA, some degree of POP was noted in 93.6% of women attending gynaecology clinic, majority of whom were asymptomatic.^1^ The prevalence of symptomatic prolapse varies widely and may be present in up to 20% cases.^2^

The definitive treatment of symptomatic prolapse or prolapse stage 2 and beyond is surgery. Among surgical options pelvic reconstructive surgery with vaginal hysterectomy is the treatment usually offered to the patients. Conservation of uterus seems pertinent in young patients and who have not completed their family. Local data revealed that 12% of patients with POP were unmarried, while 16.6% were nulliparous.^3^,^4^ Preserving the uterus in these young women with POP is a challenge. Various surgical procedures are used by surgeons to conserve the uterus. Manchester repair, sacrospinous hysteropexy and abdominal or laparoscopic hysteropexy are available options. Among these procedures sacrohysteropexy is preferred in selected patients, when uterine preservation is required.^5^ Cure rates up to 91-100% have been reported.^6^

Pelvic reconstructive surgery with vaginal hysterectomy or Manchester repair is the usual treatment which is offered to patients with prolapse stage 2 and beyond in the study set up. In an attempt to conserve the uterus, sacrohysteropexy (SHP) was introduced for the young patients with POP in 2007. This procedure is new in the study setup and for the surgeons. Therefore, we planned to review the case files of these patients to assess the outcome, safety and patient satisfaction.

## METHODS

This is a descriptive case series of patients who underwent SHP for POP. The data includes patients operated at Sind Government Lyari General Hospital, between January, 2007 to August, 2010, and Civil Hospital, between September 2010 to October 2015. Both of these are teaching hospitals of Dow Medical College and Dow University of Health Sciences, Karachi. All patients were operated by same researchers.

Inclusion criteria were women presenting with POP, aged less than or equal to 40 years, parity 0 to 4 and having normal menstrual cycles. Patients older in age, not keen to have further pregnancies or having menstrual irregularities or other pelvic pathology were excluded. Patients with significant rectocele, enterocele or stress incontinence were also excluded from study.

Patients were selected from out-patient department of Gynaecology by researchers after obtaining verbal, informed consent. Detailed history was obtained and clinical examination performed to establish diagnosis, stage of prolapse and exclude pathology like pelvic or abdominal mass and pelvic inflammatory disease. Relevant investigations were performed to identify co-morbid conditions. If present, anaemia was corrected and urinary tract infection was treated. Any medical disorders like hypertension or diabetes were controlled in collaboration with Department of Medicine. Cardiac and anaesthesia fitness was obtained. Patients were then admitted in post-menstrual phase. Routine pre-operative care was provided including informed written consent.

SHP was performed under general anaesthesia through laparotomy. A polypropylene mesh (Ethicon^®^) 6 x 11cm (trimmed to appropriate size) was placed through a retroperitoneal tunnel and sutured to anterior longitudinal ligaments above and utero-sacral ligaments (including substance of cervix) below. Prolene No 1 was used for sutures. It was adjusted so as to ensure that uterus was pulled up to its normal anatomical position without tension. Peritoneal window was then closed. Bilateral round ligament plication was considered when extra lengthening and laxity of round ligaments was evident during surgery. It was performed by using a continuous suture of No 1 Prolene from one to the other end of each round ligament and tying the ends of suture together. Laparotomy wound was then closed. Digital vaginal examination was performed to confirm that the level of cervix was above ischial spines. Any intra-operative complications like primary haemorrhage (blood loss > 500 mls or urgent need for blood transfusion), visceral injury (ureter, bowel) were noted.

Routine post-operative care was provided. Following complications were noted: secondary haemorrhage (blood loss > 500 mls or urgent need for blood transfusion in post-operative period), paralytic ileus (silent bowels 24 hours after surgery in the absence of signs of peritonitis or bowel injury), peritonitis (abdominal distension with signs of peritoneal infection), abdominal wound infection (purulent wound discharge). Patients were discharged on 3^rd^ or 4^th^ day. Abdominal stitches were removed on 8^th^ post-operative day as out-patient procedure.

Patients were called in OPD after six weeks and enquired about any complaints, like backache. They were examined to exclude recurrence and were asked about whether they were satisfied with the result of surgery.

All data entered in predesigned proforma were shifted to and analyzed using SPSS version 16. Numerical variables ie., age, BMI, parity, age of last born, haemoglobin (Hb) and days of hospital stay are presented as mean ± SD. Categorical variables eg., factors associated with utero-vaginal prolapse, it’s grade, duration of surgery, blood loss, complications of operation, success of operation, complaints on six week follow up and patient’s satisfaction with the results of procedure, are presented as frequencies and percentages. Early post-operative success of operation was defined as level of lowermost part of cervix above ischial spines on digital vaginal examination at end of procedure and on 6 week follow-up visit.

**Fig-1:**
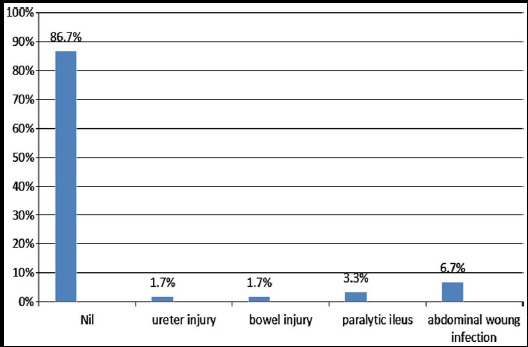
Complications of SHP.

## RESULTS

A total of 60 patients were studied with the mean age 32.6 years and parity 3. Two patients were under the age of 20 years. Four patients were unmarried and another two were nulliparous. Most frequent factor seen in association with prolapse was home delivery (n=33, 55%), followed by chronic constipation (n=28, 46.7%) and heavy weight lifting (n=21, 35%). Second stage of prolapse was seen in 34 ie., 56.7% cases, while 26 ie., 43.3% had third stage of prolapse.

Round ligament plication was performed in 40 % patients in addition to SHP. Duration of surgery was < 2 hours in 57 patients (95%), 2 to 3 hours in two and > 3 hours in one patient. This last patient sustained bowel injury which was repaired. Blood loss was negligible in majority of cases, being 100 – 200 mls only in three cases. Operation was successful in 100% cases.

**Tables-I:** Demographic Data.

*Variable*	*Mean*	*±SD*	*Minimum*	*Maximum*
Age (years)	32.6	6.14	15	40
BMI (weight in kg / height in metre^2^)	24.6	1.64	22	29
Parity	3.04	1.33	0	4
Age of last born (years)	5.35	3.16	1	13
Hb (gm/dl)	11.3	1.12	9	15
Hospital stay (days)	4.12	1.56	3	14

**Fig-2:**
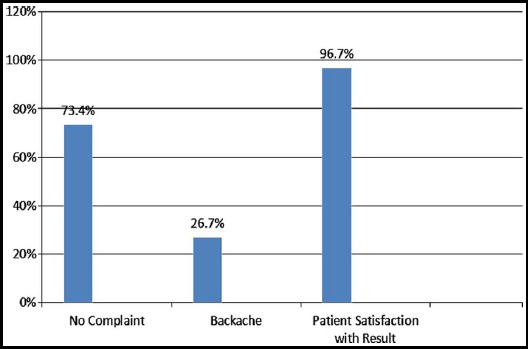
Six week follow up.

Out of all 60 cases, 52 (86.7%) did not have any complication. One patient had right ureteric damage, which was identified on second post-operative day. Her ureteric reimplantation was performed by concerned department. The post-operative result was satisfactory. One patient sustained injury to rectum, which was immediately diagnosed. Repair of rent was performed by general surgeon, along with colostomy. Patient was alright after closure of colostomy. Two patients had paralytic ileus without any signs of peritonitis. Condition resolved with intravenous fluids, electrolyte balance and antibiotics, within two days. Four patients suffered from abdominal wound infection and were treated with wound dressing, appropriate antibiotics and later resuturing. Mean duration of stay in hospital was 4 days.

Upon follow-up, except for two women who sustained visceral injury, all other patients (96.7%) were satisfied with results of operative procedure. Sixteen (26.7%) complained of backache which was relieved by nonsteroidal anti-inflammatory agents. We also want to report one case of recurrence after five years.

**Table-II:** Factors associated with UVP.

*Variable*	*n*	*%*
Home delivery	33	55
Difficult delivery	25	41.7
Instrumental delivery	8	13.3
Prolonged labour	14	23.3
Delivery of big baby	6	10
Smoking	3	5
Obesity (BMI > 25)	10	16.7
Chronic constipation	28	46.7
Heavy weight lifting	21	35
Previous pelvic surgery	6	10

## DISCUSSION

POP is a common health problem in women negatively affecting quality of life and is a leading cause of hysterectomy for benign disease.^7^ The higher prevalence of predisposing factors in developing world seems to be responsible for the increased risk of POP at an early age. In a study of POP, a prevalence of 21.2% was reported in women under 25 years in Sind province and 12.12% were unmarried young women.^3^ In comparison prevalence of POP was 1.6% in 20 -39 years age group in USA.^7^ We specially selected young patients for our study as we aimed to see the results of uterine preservation in them.

The conventional surgical treatment for POP is vaginal hysterectomy with pelvic floor repair. Uterus is removed despite being normal. Moreover, removal of uterus fails to address the aetiology of prolapse. Up to 40% of patients undergoing vaginal hysterectomy have been reported to present subsequently with vaginal vault prolapse.^8^ A two fold risk for ovarian function failure is reported in women undergoing hysterectomy as compared to women with retained uterus.^9^ With increasing awareness, larger number of women worldwide desire conservation of uterus. In a study of 220 women with intact uteri under evaluation for POP, 60% wanted to avoid hysterectomy if acceptable alternative was available.^10^

Young patients need uterine preservation. Conservation of uterus not only supports the pelvic floor, it preserves fertility, improves sexual function and wellbeing. It decreases the risk associated with hysterectomy. It is performed in less time.

The earliest uterine conserving surgery performed was Manchester repair.^7^ It is not being favored nowadays due to its association with subfertility and obstetric complications. Transvaginal sacropinous fixation is another option, but due to close proximity of sciatic nerve and pudendal vessels and nerve to sacroscopious ligament, this surgery may lead to significant buttock and leg pain and haemorrhage.^11^ Ventrosuspion is technically simple but high recurrence rate of prolapse refute its practical application. A study reported that eight women out of 9 who underwent ventrosuspension had recurrence within three months.^12^ Uterosacral plication is reported to gives better results than ventrosuspension. In a case series of seven women, Wu MP found no recurrence of prolapse at 9-17 months follow up.^13^ Maher reported success rate of 79% in a series of 43 women at 12 months follow up.^14^ But the procedure is associated with complications including massive haemorrhage, buttock pain and recurrent cystocele. Stepp and Paraiso reported ureteral injury after utero sacral plication.^15^ In one of our patients, who was young and obese with BMI above 25 kg/m^2^, we performed uterosacral plication concomitant with SHP. She ended up having prolonged post-operative pain and vomiting. Right ureteric injury was diagnosed and re-implantation of ureter was performed for her.

SHP results in satisfactory anatomy and functional result with normal vaginal axis. It involves the basic principle of elevating the uterus and suspending it to sacrum using a mesh.^16^ Several variations of this procedure have been described. Cutner et al performed SHP by passing Marceline tape through uterosacral ligaments to re-suspend the uterus to sacral promontory bilaterally.^17^ Price N used polypropylene bifurcated ‘Y’ shape mesh, between sacrum and anterior surface of cervix.^8^ Massey F also used polypropylene mesh, but sutured the lower end on posterior cervix at the level of utero-sacral ligaments.^18^ We used a rectangular piece of polypropylene mesh in our patients. It was stitched to the uterosacral ligaments and cervix below and to sacral promontory above. Use of mesh may be associated with the risk of infection and intrusion of mesh from the vagina. Literature reveals studies where extrusion of mesh was reported. In our setting we decided to use mesh because we thought it would give us better results. Price N^8^ did not report any case of erosion, infection or rejection of mesh in their series, nor did we encounter this complication in our patients. Api M performed this surgery laparoscopically using a different ‘Cravat’ technique.^11^ Robotic SHP has also been reported to provide results comparable to abdominal SHP.^19^

Sumaira did not report any visceral injury in their study.^4^ This differs from a frequency of 3.4% in our study where one patient sustained rectal injury which was identified and operated immediately. Another patient suffered ureteric injury as discussed earlier. Moity FM and colleagues studied 33 cases of SHP, of whom one patient suffered rectal injury.^20^

The duration of surgery was less than two hours in 93.3% of the patients which is significantly longer than less than one hour reported by Moity and Karim.^20^,^21^ At 6 weeks post-operative follow up, 96.7% of our patients were satisfied with the surgery. This is consistent with 96.9% satisfaction rate reported by Api M.^11^ We judged patients satisfaction on clinical basis i.e correction of prolapse, severe pain, backache or severe post operative complications. Minor degree of post operative complaints are usually not taken significant by patient. For patients under study, their greatest achievement was cure of problem with restoration of uterus.

A limitation of our study was lack of long term follow up. Unfortunately in the community where study was conducted majority of patients abscond, they came for visit only in case of some complaint. Hence we did not include long term follow-up of patients in the objectives of this current review. But we have noted one patient with recurrence of prolapse after five years of SHP. She is a nulliparous obese lady with history of chronic constipation. The cause of recurrence could also be congenital pelvic floor weakness. Recurrence rate of 16.7% has been reported earlier in a local study.^4^ Api M et al. followed their cases up to a median of 23.9 months and did not find any case of recurrence.^11^

## CONCLUSION

In community where study was conducted due to lack of education and financial reason most of the patients do not come for regular follow up. However they do come if they develop any post-operative complaint. Nonetheless, early post-operative success was 100%. It is a safe procedure for the young women with POP, provided it is done carefully. It is to be noted that the two incidences of trauma occurred earlier when the procedure was introduced. With increasing number of surgeries being performed, no such incidence was reported.

As this study is a retrospective review of patients who had SHP, hence authors could only judge patients’ satisfaction from information available. In future, specified instrument such as SF-12 or PSQ-18 should be used to assess patient satisfaction.
